# The Heavy Load of Lead: Ergonomic Stress Heightens Exposure-Related Neuropathy

**Published:** 2005-12

**Authors:** Dinesh C. Sharma

Long-term lead exposure among industrial workers can result in neuropathy (a disorder of the peripheral nervous system), while lower exposure levels cause muscle weakness. Until recently, however, the interaction between lead toxicity and chronic repetitive muscle use had not been investigated. Researchers from the Center for Occupational and Environmental Neurology in Baltimore now report that the impact of chronic lead exposure is augmented by concomitant ergonomic stress **[*EHP* 113:1730–1734]**.

The study included 80 lead smelter workers who were routinely exposed on the job to inorganic lead dust and (to a lesser extent) lead fumes. Historical blood lead records for all the workers were available from the smelter, which checked all employees’ blood lead at least quarterly. These records showed that workers had high chronic exposure in the distant past, much lower exposure in the more proximate past, and still lower exposure at the time of the study. The researchers also measured current blood and bone lead levels and used the historical records to calculate two metrics of cumulative lead exposure—working-lifetime integrated blood lead (IBL) and working-lifetime weighted-average blood lead (TWA).

The team used the current perception threshold test to examine nerve fiber populations in the workers’ shoulders, arms, wrists, and hands. This test measures the amount of electrical current needed to induce a sensation. The team also created a three-tiered ergonomic stress rating based on all the different jobs the workers had ever performed, cumulated over their employment history. This was used to arrive at a time-weighted average ergonomic stressor. Sensory nerve conduction threshold was measured in large myelinated, small myelinated, and unmyelinated nerve fibers.

The results showed that decrements in nerve function—a precursor to neuropathy—were limited to large and small myelinated sensory nerve fibers, with a threshold effect at a TWA of 28 micrograms per deciliter. At higher levels of lead exposure and presence of ergonomic stress, nerve fibers were more susceptible to increased damage, something that has never before been shown in human studies. The investigators suggest that nerves affected by lead are more susceptible to traction or mechanical compression, as would occur in the carpal tunnel of workers who perform activities such as heavy lifting and shoveling.

Measures of chronic lead exposure may serve as strong predictors of impaired nerve function. In addition, the authors believe they have been able to separate the impact of two components of cumulative blood lead—duration and intensity—with exposure intensity appearing to have a greater influence than duration on the outcome studied. Finally, the authors point out that although TWA and IBL are associated with peripheral nerve damage, bone lead—another measure of chronic exposure—is a weak predictor of lead effects in the nervous system because it reflects only that lead stored in the bone compartment and not necessarily the cumulative blood lead to which peripheral nerves were exposed.

## Figures and Tables

**Figure:**
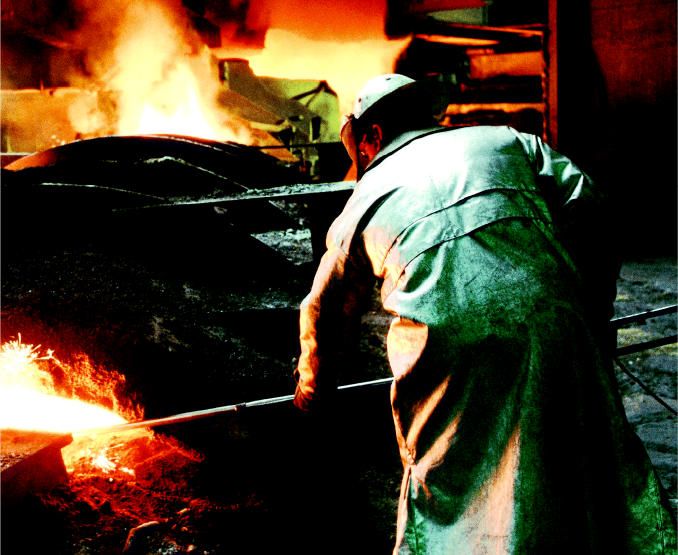
Double jeopardy. Ergonomic stress may heighten the threat posed by on-the-job lead exposure.

